# A global biogeographic regionalization for butterflies

**DOI:** 10.1098/rstb.2023.0211

**Published:** 2025-01-09

**Authors:** Collin P. Gross, April M. Wright, Barnabas H. Daru

**Affiliations:** ^1^ Department of Biology, Stanford University, Stanford, CA 94305, USA; ^2^ Department of Biological Sciences, Southeastern Louisiana University, Hammond, LA 70402, USA

**Keywords:** biogeographic regionalization, butterflies, phylogenetic beta diversity, lepidoptera

## Abstract

The partitioning of global biodiversity into biogeographic regions is critical for understanding the impacts of global-scale ecological and evolutionary processes on species assemblages as well as prioritizing areas for conservation. However, the lack of globally comprehensive data on species distributions precludes fine-scale estimation of biogeographical regionalization for numerous taxa of ecological, economic and conservation interest. Using a recently published phylogeny and novel curated native range maps for over 10 000 species of butterflies around the world, we delineated biogeographic regions for the world’s butterflies using phylogenetic dissimilarity. We uncovered 19 distinct phylogenetically delimited regions (phyloregions) nested within 6 realms. Regional boundaries were predicted by spatial turnover in modern-day temperature and precipitation seasonality, but historical climate change also left a pronounced fingerprint on deeper- (realm-) level boundaries. We use a culturally and ecologically important group of insects to expand our understanding of how historical and contemporary factors drive the distribution of organismal lineages on the Earth. As insects and global biodiversity more generally face unprecedented challenges from anthropogenic factors, our research provides the groundwork for prioritizing regions and taxa for conservation, especially with the goal of preserving the legacies of our biosphere’s evolutionary history.

This article is part of the discussion meeting issue ‘Bending the curve towards nature recovery: building on Georgina Mace's legacy for a biodiverse future’.

## 1. Introduction

Biogeographic regions describe the spatial distribution of life on the Earth and form the basis of many studies in ecology, conservation and evolutionary biology [1–3]. Biogeographic regionalization involves dividing the Earth’s surface into distinct regions based on shared ecological and evolutionary characteristics. These regions are characterized by unique assemblages of species, ecological communities and ecosystems. Understanding biogeographic regions is crucial across the fields of ecology, evolutionary biology and conservation because it allows the investigation of factors driving biodiversity patterns and ecosystem dynamics across ecological scales [4–6], and the possible changes in these factors in the past and into the future. Thus, delineating biogeographic regions can enable the identification of the underlying processes that shape species composition, community structure and ecosystem functioning [7] as well as enable the prediction of how contemporary factors such as habitat loss and climate change may alter these regions, and delineate areas and species for conservation priority [8–11].

Combining biogeographic regionalizations with phylogenetic estimation allows researchers to account for the role of evolutionary history of regional biotas in shaping species distributions. For instance, areas with extensive local sympatric speciation or endemic radiations are likely to be more evolutionarily distinct from surrounding regions, potentially forming a separate phylogeographic cluster. On the other hand, in cases where speciation occurs in allopatry, especially due to geographical isolation, the separation of biogeographic clusters is less clear because sister species can occur on either side of a regional boundary [7]. Such knowledge can serve as a crucial step in addressing spatial evolution of biodiversity and identifying important units for conservation.

In terrestrial ecosystems, global biogeographic regionalizations have focused on vertebrates [1,12] or plants [13]. For instance, Holt and colleagues [1] updated Wallace’s [3] zoogeographic regions using geographical distributions and phylogenetic relationships of the world’s mammal, bird and amphibian species and divided global land areas into 20 regions nested into 11 larger realms, which are still widely used today [12,14,15]. However, these regionalizations rely heavily on globally comprehensive species geographical information, limiting the number and types of taxa that can be analysed. This information is not readily available for ecologically and economically invaluable invertebrate taxa, such as butterflies, which lack distribution data at a global scale [16–18]. Additionally, examining the patterns and drivers of regionalization schemes across disparate terrestrial taxa aids us in understanding the historical and modern-day processes that govern the broad-scale distribution of life on our planet.

Butterflies (Lepidoptera: Papilionoidea) are thought to have originated in the middle Cretaceous (102.5–100 Ma) in North or Central America [19,20]. Since then, they have diversified into approximately 19 000 extant species [21], play key ecosystem roles as pollinators (as adults) and herbivores (in their larval form) and are involved in a wide array of ecological and coevolutionary interactions ranging from myrmecophily to phytochemical sequestration [22–25]. Despite their ecological, cultural and evolutionary importance, no attempt has been made to incorporate phylogenetic structure into our understanding of butterfly regionalization, although recent work has illuminated the group’s global phylogeographic history [19]. Comprehensive regionalization is hindered in part by a lack of comprehensive global distribution data at the species level. The country-level occurrence database compiled by Pinkert *et al*. [21] remained the only available dataset on global butterfly distributions until now [26]. Previous studies of butterflies have primarily focused on regional or continental assessments or have concentrated on specific clades [4,27–30], but these have largely overlooked their evolutionary history and how this history underlies modern distributions.

Here, using an unprecedented dataset including native range maps for 10 372 butterfly species, representing about 53% of global butterfly diversity along with their phylogenetic relationships, we provide the first global phylogenetic biogeographic regionalization (“phyloregions”) of butterfly assemblages. Specifically, we ask (i) what are the global phylogenetically delimited biogeographic regions for butterflies? (ii) what are the determinants of biogeographical boundaries for butterflies? (iii) how do biogeographic boundaries for butterflies compare with those observed in other terrestrial taxonomic groups? We hypothesized that butterfly phyloregions would be relatively congruent with those previously published for terrestrial plants and tetrapods [1,12,13]. In particular, because of butterflies’ coevolutionary relationships with flowering plants [23–25], we expected butterfly and angiosperm phyloregions to be more congruent than those of butterflies and tetrapods. As terrestrial ectotherms, we also expected butterfly regionalization to largely be driven by global temperature patterns [31].

## 2. Methods

### (a) Source data

To begin our phylogenetic regionalization scheme for butterflies, we used a new set of native range polygons for butterflies created by Daru [26]. Briefly, these polygons are modelled distributions based on point data available from the Global Biodiversity Information Facility (GBIF [32]), clade-specific constraints on dispersal [33] and species distribution models using maximum entropy. To reduce the effects of uneven or biased sampling, we spatially thinned point records for species with at least five unique localities and ensured that no two points were within 30 km of one another to generate alpha hull polygons [34–36]. To facilitate scaling across a large number of species with very few occurrences, we converted alpha hull polygons into pseudo-occurrence records by first converting to a raster, converting raster grids to points and thinning points to a maximum of 500 records per species to reduce spatial autocorrelation [13,37,38]. Each species range map was stacked at the resolution of 100 km × 100 km grid cells to generate a presence and absence matrix of 10 372 species in 13 316 grid cells using the *polys2comm* function in the R package phyloregion [39].

### (b) Phylogeny

To examine how butterfly lineages are regionalized across the globe, we used a time-calibrated tree of 2254 species from Kawahara *et al*. [19] representing all families, subfamilies and tribes, and 92% of described genera. To complete the tree, we grafted the remaining species that were not in the original tree onto the backbone topology using the function *phylo.maker* in the package V.Phylomaker2 under scenario 2 [40]. In this scenario, new tips are bound to randomly selected nodes at and below the genus-level (if congeners exist in the backbone; *n* = 8 293 species added in this way) or family-level (if congeners do not exist in the backbone; *n* = 401 species added in this way) basal node. We created 100 random trees based on this method to build a consensus phylogenetic regionalization scheme for butterflies.

### (c) Data analysis

#### (i) Building regionalization schemes

To quantify spatial turnover in butterfly assemblages, we first calculated species-level beta diversity using the matrix of Simpson pairwise dissimilarities across all 100 km × 100 km grid cells using the *beta_diss* function in the R package phyloregion [39]. We then calculated phylogenetic beta diversity among grid cells using the Simpson-derived pairwise phylogenetic dissimilarity matrix using the function *phylobeta* for each of our 100 trees and used the average dissimilarity matrix for downstream analyses.

To map biogeographic regions, we used the function *optimal_phyloregion* to determine the optimal number of biogeographic regions for both species-level and phylogenetic beta diversity matrices based on the elbow method, which calculates the optimal number of clusters based on explained variance and internal consistency of clusters [41]. We then determined the best hierarchical clustering algorithm to use in building our biogeographic regionalization schemes by using the function *select_linkage*, which compares eight commonly used clustering algorithms based on their cophenetic correlation coefficient, a measure of how well a dendrogram preserves Simpson distances from the original distance matrix. For both our phyloregions and species bioregions, this coefficient was highest for the unweighted pair-group method with arithmetic averages (UPGMA) method. Next, we examined the deeper relationships between regions by cutting the dendrogram at a depth that preserved at least 60% of the variation in phylogenetic beta dissimilarity to produce a set of phylogenetically delimited biogeographic realms with the phyloregions nested in them. This cut-off threshold consistently produced realms that were similar in shape and number to those previously established for other taxa [1,12,13,42].

To better characterize each region in terms of species and phylogenetic diversity, we computed the total species richness and phylogenetic diversity (PD, the sum of tree branch lengths in a community phylogeny; [43]) for each region. We also calculated the mean regional weighted species (species richness, with each species inversely weighted by the number of grid cells it occupies; [44]) and phylogenetic endemism (phylogenetic diversity, with branch lengths inversely weighted by the range size of their descendent clades; [45]) by averaging endemism values across all grid cells within the region, allowing us to determine which regions had larger numbers of species with small ranges rather than the number of species endemic to a whole region. We also computed the mean value of phylogenetic beta diversity between a focal phyloregion and all other phyloregions in the study area as a measurement of the evolutionary distinctiveness of each phyloregion.

To confirm the robustness of our regionalization schemes, we repeated these analyses eight times: once using the UPGMA algorithm with the subset of butterfly species shared between our dataset and the original Kawahara *et al*. tree ([19]; *n* = 1691), again using the UPGMA algorithm with all of our butterflies at the genus level (*n* = 1600) and once on the full species dataset, each with six additional clustering algorithms: (1) centroid linkage clustering (unweighted pair-group method with centroids; UPGMC), (2) median linkage clustering (weighted pair-group method with centroids;WPGMC), (3) weighted pair-group method with arithmetic averages (WPGMA), Ward clustering with (4) and without (5) Ward’s clustering criterion and (6) maximum (complete) lineage clustering.

#### (ii) Comparing butterfly phyloregions with other taxa

To assess the congruency between butterfly phyloregions and those of other terrestrial clades, we used the V-measure (a measure of how homogeneous and complete clusters are) approach [46] on six previously published phylogenetic regionalization schemes for plants [13], reptiles [12], amphibians, birds, mammals, and combined amphibians, birds and mammals at the region level [1]. This approach compares two independent regionalization schemes and calculates a score (V-measure) between 0 (no association between compared regionalizations) and 1 (perfect association). For example, the V-measure score between butterflies and another taxon would decrease if single butterfly regions overlapped with multiple separate regions of the other taxon and *vice versa*. To test whether V-measures were significantly different from a random expectation, we compared the observed measures with a random distribution of measures created from 999 random regionalization schemes with the same number of regions as the scheme of comparison. We created random schemes by first randomly sampling a number of terrestrial cells corresponding to the number of random bioregions needed, then creating Voronoi polygons from those points using the *voronoi* function in the R package terra [47] and finally cutting the Voronoi polygons using the outline of global landmasses. We used the same approach to quantify the congruence between our phyloregions and species bioregions, genus-level phyloregions, phyloregions derived from the Kawahara *et al*. [19] subset and phyloregions and realms defined by the six additional clustering algorithms.

To assess the pairwise correlations of boundaries across different phylogenetic regionalization schemes, we first calculated the distance of each 100 km × 100 km grid cell from a boundary in each scheme. We then used a modified version of a *t*-test that is appropriate to test for correlations among spatial processes [48] to test whether the distance of each cell from a boundary is similar among the schemes. If two phylogenetic regionalization schemes are similar, boundaries cross the same areas of the planet, and any given cell shows a similar distance from the boundary in both schemes. Prior to testing, we log-transformed distance values in km.

#### (iii) Examining predictors of phyloregion boundaries

We examined the predictors of boundaries between butterfly phyloregions using a simultaneous autoregressive (SAR) spatial modelling approach, which accounts for spatial autocorrelation when modelling the relationships between boundaries and predictors [49,50]. Four predictor variables (derived from the WorldClim database; [51]) represented spatial climate heterogeneity, defined as the coefficient of variation between each cell and eight adjacent cells: temperature seasonality, precipitation seasonality, mean annual temperature and mean annual precipitation. Two additional variables included climate change velocity since the last glacial maximum (LGM) and terrain ruggedness index (TRI; mean absolute difference in elevation between each cell and eight adjacent cells). We centred all predictor variables on a mean of zero and scaled to a standard deviation of one. All predictors showed variance inflation factors of less than two, indicating minimal collinearity.

For our SAR modelling approach, we built a hierarchical generalized linear model (HGLM), in which the response variable was a binomially distributed binary variable determining whether a given cell is in contact with a phyloregion boundary or not. This model accounted for spatial autocorrelation among cells’ predictor values using neighbourhood matrices in the error term. We used a neighbourhood size of 282.84 km, which was the maximum distance required for all cells to be connected to at least one other, and limited our dataset to only the cells within 200 km of either side of a boundary to avoid excessively zero-inflated data. We repeated this analysis three times: once for all boundaries, once for only the deep boundaries between realms and once for only the shallow boundaries within realms. We report the effect size of each variable as Fisher’s *z*, a measure independent of sample size that enables robust comparisons of effects estimated across different models.

#### (iv) Identifying clades driving realm-level divisions

Finally, we were interested in the butterfly clades that were key drivers of faunal turnover across realm boundaries. To identify such ‘indicator clades’, we used a node-based approach [52]. First, we identified nodes in the global butterfly phylogeny whose daughter lineages had minimal overlap in their geographical distribution, defined as a Geographic Node Divergence score (GND) greater than 0.65. For each of these nodes, we identified the grid cells in which daughter lineages occurred and calculated a specific overrepresentation score (SOS) for each cell, with more positive scores indicating overrepresentation of one daughter lineage, more negative scores indicating overrepresentation of the other daughter lineage and scores closer to zero indicating equal representation of both lineages. We then modelled SOS values as a function of cell realm membership for each of 15 realm pairs to identify which of the high-GND clades contributed to differences in the butterfly faunas of paired realms, focusing only on comparisons between positive and negative mean SOS values. The contribution of clades to realm division was measured by the *R*^2^ value of each model.

## 3. Results

### (a) Biogeographic regions for butterflies

**Figure 1.**
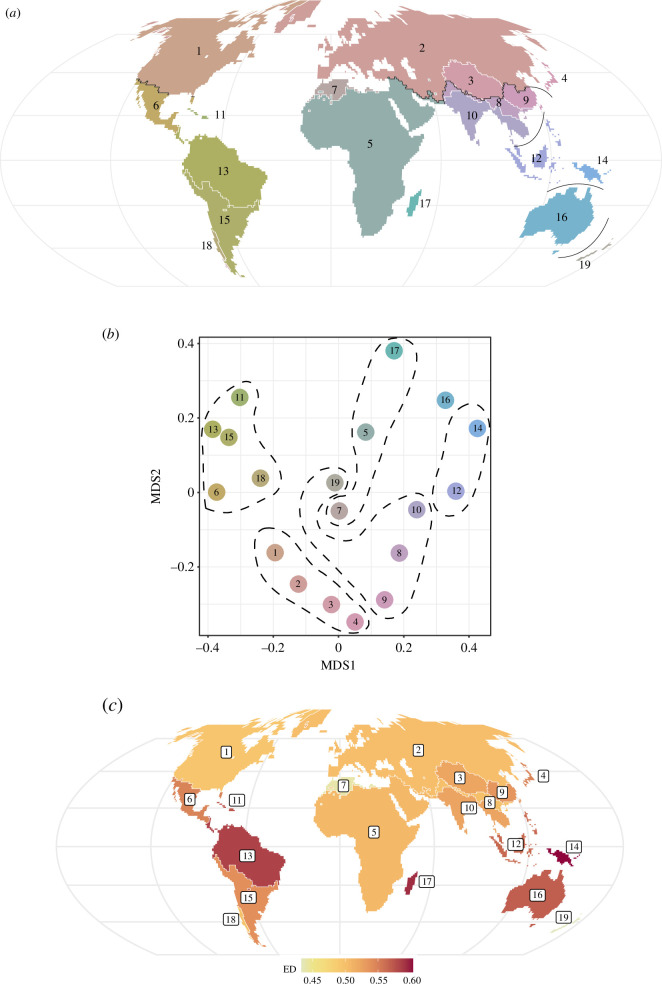
A global phylogenetic regionalization of butterfly species (*n* = 10 372 species). (*a*) Map showing the 19 major butterfly regions around the world, defined by phylogenetic similarity. White lines represent divisions between regions (80% of variation in phylogenetic dissimilarity explained), while black lines represent deeper divisions between realms (65% of variation explained). (*b*) Non-metric multidimensional scaling (NMDS) plot showing relationships between regions (numbered circles) and realms (dotted outlines). (*c*) Regions coloured by evolutionary distinctiveness (ED). A region is darker if the mean value of phylogenetic beta diversity between it and all other regions is greater. Numbered regions: 1) North American, 2) Eurasian, 3) Tibetan, 4) Japanese, 5) Afrotropical, 6) Mesoamerican, 7) Maghrebi, 8) Hengduan–Himalayan, 9) Chinese, 10) Indian, 11) Caribbean, 12) Malesian, 13) Amazonian, 14) Papuasian, 15) Pampeo-Andean, 16) Australian, 17) Madagascan, 18) Valdivian and 19) Novozelandic. Maps are Mollweide projections.

Using the ‘elbow’ method, we identified 19 clusters based on shared phylogenetic affinity (phyloregions) and 13 regions based on species beta diversity (figure 1*a*, electronic supplementary material, figure S1) that explained 80% and 68% of the variation in beta diversity, respectively. The boundaries between species and phylogenetic regionalization schemes were markedly similar (V-measure = 0.84, *p* < 0.001). Hierarchical clustering and nonmetric multidimensional scaling (NMDS) analyses revealed that deep divisions between six clusters (realms) accounted for 65% of the variation in phylogenetic beta diversity across grid cells (figure 1*b*): Holarctic (North American, Eurasian, Tibetan, Japanese), Indo-Malayan (Hengduan–Himalayan, Chinese, Indian, Novozelandic), African (Afrotropical, Maghrebi, Madagascan), Nusantaran (Malesian, Papuasian), Australian and Neotropical (Mesoamerican, Caribbean, Amazonian, Pampeo-Andean, Valdivian).

Given that 83% of the species in our dataset were grafted onto the backbone phylogeny, it is worth noting how the species in the original Kawahara *et al*. [19] tree are distributed among the regions. We found that although no region had more than 50% of its species present in the backbone, the Japanese region was most represented—with 49% of its species in the backbone—followed by the Caribbean and Valdivian regions, for which 37% and 34% of species were represented, respectively (electronic supplementary material, figure S2). In contrast, the Novozelandic, Afrotropical, Madagascan and Papuasian faunas were most underrepresented, with 13%, 13%, 15% and 16% of species in the original backbone, respectively (electronic supplementary material, figure S2). This is particularly striking in the case of the Afrotropical realm, where just 290 of its 2301 species were present in the original tree.

**Figure 2.**
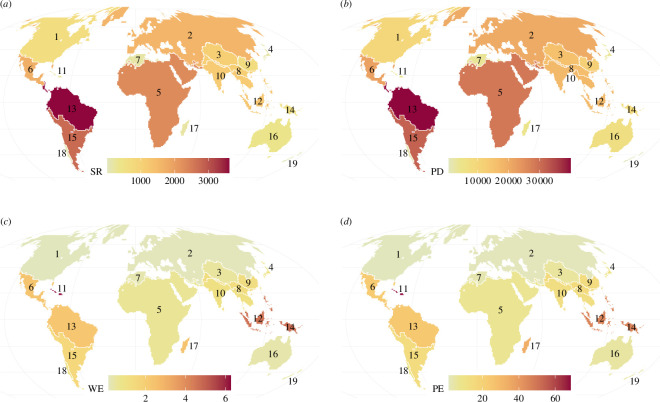
Diversity and endemism in the 19 major butterfly regions around the world. Species richness (*a*) and phylogenetic diversity (Faith’s PD; (*b*) are calculated across all species in each region. Weighted species (*c*) and phylogenetic endemism (*d*) are averaged across 100 km × 100 km grid cells within a region.

We found that the Papuasian, Madagascan and Amazonian phyloregions were the most evolutionarily distinct, each with mean phylogenetic beta diversity of 0.60, 0.59 and 0.58, respectively (figure 1*c*). The most species-rich and phylogenetically diverse regions were the Amazonian, Pampeo-Andean and Afrotropical (figure 2*a,b*), and the regions with the highest average endemism were the Caribbean, Papuasian and Malesian (figure 2*c,d*).

Phylogenetic regionalization using only the species in our dataset that are found in the Kawahara *et al*. [15] tree produced 13 regions that together explained 81% of the variation in phylogenetic beta diversity across cells and 5 realms that explained 64.3% of the variation (electronic supplementary material, figure S3). Our full phylogenetic regionalization scheme was robustly supported by this limited dataset, with high congruence between the schemes (V-measure = 0.78, *p* < 0.001). The genus-level scheme produced 12 regions that explained 81% of the variation in phylogenetic beta diversity and with a lower V-measure score (0.75, *p* < 0.001; electronic supplementary material, figure S4).

Across the clustering algorithms we used to create phyloregions, the Maghrebi–Afrotropical boundary and the northern border of the Hengduan–Himalayan region were consistent features, and distinct Madagascan and Papuasian regions were features of 6/7 algorithms (electronic supplementary material, figure S3 and S5). The Tibetan region appeared across 5/7 algorithms, and the Andeo-Pampean-Amazonian boundary appeared in 4/7 algorithms. Some form of boundary was generated by 4/7, 4/7 and 5/7 algorithms between eastern and western North America, between the wet and dry tropics of southern Africa, and between northern and southern Australia, respectively. V-measure scores between the 7 clustering algorithms were greater than 0.6 for both regions and realms (electronic supplementary material, tables S1 and S2), and spatial correlation coefficients were high (greater than 0.8) among clustering algorithms for borders between regions and realms (electronic supplementary material, tables S3 and S4), indicating strong support overall for our regionalization scheme.

### (b) Similarity of phyloregions across taxonomic groups

We contrasted the boundaries of our butterfly phyloregions with those of other terrestrial taxonomic groups using the V-measure method [46], which examines the intersections among regions of butterflies and other taxa. We found high similarity between butterfly phyloregions and those of other taxonomic groups, with all pairwise comparisons (V-measure scores) significantly higher than 0.7 (table 1). However, butterfly phyloregions showed the highest congruence with tetrapod phyloregions (V-measure = 0.77; *p* < 0.001). Within tetrapods, regions defined by reptile clades were most similar to those defined by butterflies (V-measure = 0.76, *p* < 0.001). Plant-defined phyloregions were least congruent (V-measure = 0.71, *p* = 0.002), largely driven by differences in the temperate Southern Hemisphere, the Mediterranean and Central Asia (electronic supplementary material, figure S6).

**Table 1.** Comparing regions defined by other clades with those defined by butterflies using V-measure scores and correlation coefficients from spatially explicit *t*-tests. Higher V-measure scores indicate higher congruency between regional polygons for butterflies and the focal taxon; higher correlation coefficients indicate that the boundaries of regions defined by the two taxa are closer in space.

taxon	V-measure score	V-measure *p*-value	spatial correlation	spatial correlation *p*-value
all tetrapods	0.770	0	0.648	4.91 × 10^−42^
reptiles	0.763	0	0.453	6.65 × 10^−23^
birds	0.751	0	0.644	1.16 × 10^−40^
amphibians	0.741	0	0.526	8.65 × 10^−34^
mammals	0.736	0	0.641	8.78 × 10^−44^
plants	0.711	0.002	0.665	2.12 × 10^−33^

When we compared the relative positions of butterfly phyloregion boundaries with those of other taxa, we again found significant correlations between the positions of these boundaries (table 1), but the strongest correlation was between butterfly and plant regional boundaries (correlation = 0.66, *p* < 0.001), while butterfly and reptile regional boundaries showed the weakest correlation (correlation = 0.45, *p* < 0.001).

### (c) Determinants of butterfly biogeographic boundaries

**Figure 3.**
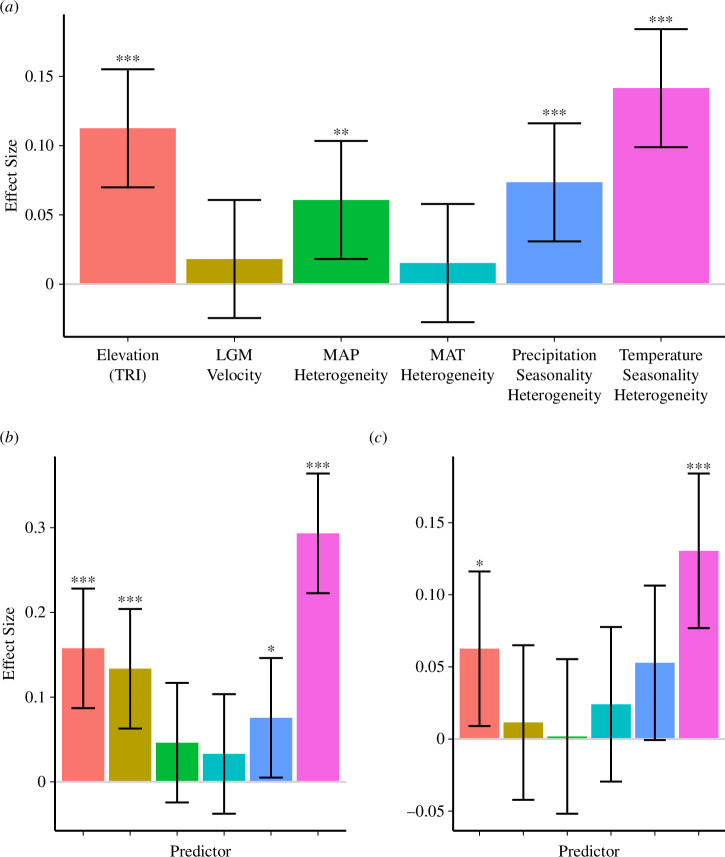
Effect sizes from spatially explicit hierarchical generalized linear models of whether a 100 km × 100 km grid cell was in contact with (*a*) any border between butterfly phyloregions, (*b*) deep boundaries between realms or (*c*) shallow regional boundaries within realms. Terrain ruggedness index (TRI) represents the mean absolute difference in elevation between each cell and eight adjacent cells; climate heterogeneity represents the coefficient of variation between each cell and eight adjacent cells. Bars indicate effect sizes (Fisher’s *z*); error bars represent 95% confidence intervals of *z*. Asterisks represent effect sizes significantly different from zero; 0 < *p* < 0.001: ***; 0.001 < *p* < 0.01: **; 0.01 < *p* < 0.05: *.

Our spatially explicit HGLM assessing the factors related to the presence of all biogeographic boundaries for butterflies showed that boundaries were more likely to occur within regions of high TRI (mean absolute difference in elevation between each cell and eight adjacent cells) and turnover in temperature seasonality and precipitation (figure 3*a*). The strongest of these effects was turnover in temperature seasonality (*z* = 0.14, *p* < 0.001). Climate change velocity since the LGM and mean annual temperature heterogeneity were not significant predictors across all boundaries.

Closer inspection of deep (between-realm) boundaries revealed that TRI, turnover in temperature seasonality and climate change velocity since the LGM were all strongly predictive of boundaries (figure 3*b*). Analysis of only shallow (within-realm) boundaries again showed that TRI and turnover in temperature seasonality were the two strongest predictors of boundaries (figure 3*c*).

### (d) Clades driving realm-level divisions

**Figure 4.**
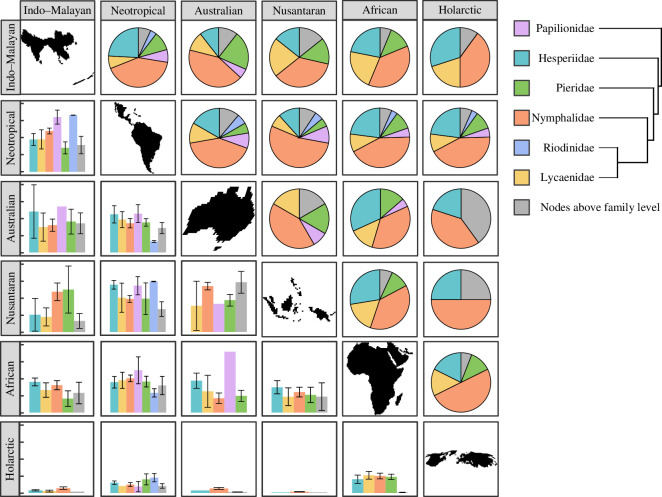
Contributions of 83 potential ‘indicator’ butterfly clades to divisions between 6 biogeographic realms, totaling 411 pairwise comparisons. Colours represent nodes within families (including between and within subfamilies, tribes and genera) or older nodes above the level of family. Above the diagonal, each pie chart represents the relative contribution of each type of node to distinguishing between the pair of realms. Below the diagonal, bars indicate the mean *R*^2^ value in a model of specific overrepresentation score (SOS) response to realm identity across all the nodes within a group for each realm pair (electronic supplementary material, table S5). *Y*-axis tick marks correspond to 0.0, 0.25, 0.50, 0.75 and 1.00. Hedylidae, sister to Hesperiidae, is not shown because neither nodes within the family nor the node subtending Hesperiidae and Hedylidae contributed to divisions between realms.

Clades with high contribution to realm divisions (‘indicator clades’) varied among realms but appeared across the butterfly phylogeny (figure 4). The median age of clades with the greatest contribution to realm-level division was 34.6 Ma, but these nodes ranged from the earliest division between Papilionidae and all other butterflies (100 Ma) to divisions within Hesperiid and Nymphalid tribes (6.9–11.6 Ma; electronic supplementary material, figure S7). The Australian and Nusantaran realms were delineated from all other realms by the oldest nodes, while the Holarctic and African realms were delineated by the youngest nodes (electronic supplementary material, figure S7). The basal node in our tree was especially important for distinguishing the Nusantaran and Indo-Malayan realms from all other realms except the Holarctic (average *R*^2^ = 0.605). Within families, the greatest contributions to divisions were by nodes within Papilionidae, especially between the Nusantaran and other realms (average *R*^2^ = 0.776). The Nusantaran realm was also distinguished from the Neotropical by clades within Riodinidae (Nemeobiini versus Euselasiini, *R*^2^ = 0.745; Nemeobiinae versus Riodininae, *R*^2^ = 0.736). The clade subtending the Riodinidae and Lycaenidae additionally had a relatively high contribution to the distinctiveness of the Neotropical realm (average *R*^2^ = 0.414).

## 4. Discussion

Overall, butterfly phyloregions shared many similarities with those published for other terrestrial taxa [1,12,13], including distinct North American, Eurasian, Australian, Madagascan, Novozelandic and Afrotropical regions. However, we found some notable differences. For instance, we found a separate realm including the islands of maritime Southeast Asia and New Guinea (the Nusantaran realm) that was distinct from the rest of the Sunda Shelf and Australia, a distinction not present in any published phylogenetic regionalization scheme for other taxa. While our Nusantaran realm was particularly distinct for its clades of swallowtail butterflies (Papilionidae, figure 4), Southeast Asia as a whole is believed to be the geographical origin of much of the world’s butterfly diversity, particularly brush-footed butterflies (Nymphalidae; [19,53,54]), and the high concentration of islands and history of sea level change in this region likely contribute to the evolutionary distinctiveness and high endemism of the region ([55–57]; figures 1 and 2). These patterns may also help to specify the centre of diversity in the region believed to be the source of diversity in surrounding areas; other studies have attempted to understand the geographical origins and dispersal of butterfly biodiversity [19,53] but used an *a priori* Wallacean [3] regionalization scheme to constrain their analyses. Our phylogenetic regionalization scheme can thus be further used to understand the geographical sources and sinks of butterfly diversity worldwide.

Like other terrestrial regionalization schemes, we uncovered distinct Novozelandic and Saharan (Maghrebi) phyloregions, although these were among the least stable across algorithms and datasets (electronic supplementary material, figures S1, S5 and S4). The native butterflies of New Zealand are unusual among other taxa from the archipelago in that they do not represent recent endemic radiations, paleoendemic relics or close affinities with other Oceanian biotas [58–61]; of the only 16 Novozelandic species in our dataset, 11 were endemic and 4 (*Argyrophenga, Dodonidia, Erebolia* and *Percnodaimon*) represent endemic genera. The low species richness and low phylogenetic endemism in our dataset likely contributed to the relatively low distinction of the Novozelandic region, which grouped within the Indo-Malayan and Holarctic realms across datasets and algorithms. Also of note here is that only 2 of the 16 Novozelandic species in our full list were included in the original backbone phylogeny [19], making this area of the world particularly subject to variation among the random trees we generated (electronic supplementary material, figure S2). The Maghrebi region—which in our dataset contains 177 species, of which 10 are endemic—jumps affinities between Afrotropical, Eurasian and Turanian regions, likely owing to a high proportion of shared lineages between these regions.

Butterfly regions had significantly high congruence with all the regions defined by other taxa that we investigated (table 1). Butterflies and plants shared the lowest V-measure scores, which was surprising given the coevolutionary relationships between butterflies and larval host plants [23–25,62], but this V-measure was only 0.059 less than the highest V-measure (butterflies and tetrapods). These values may all be relatively high because we have defined regions at the global scale at a relatively low phylogenetic beta diversity cut-off, which glosses over finer distinctions between biotas at sub-regional scales. Although there are noticeable differences in regionalization schemes among taxa, at the level at which we have defined regions, larger global-scale processes are the primary architects of discrete regions, which tend to be common across taxa [12,14]. Were we to focus on sub-regional clusters, we anticipate much higher variation in pairwise V-measures between butterflies and other taxa, as the processes occurring at these smaller spatial scales are more idiosyncratic and more difficult to generalize across taxonomic groups [12,63].

Spatial correlations between boundaries seem to be a better descriptor of similarity between regionalizations than V-measures in our analyses, showing that butterfly and plant boundaries were most highly correlated (table 1). This was unsurprising given the coevolutionary relationships between butterflies and their larval host plants [23–25,62], even though codiversification and constraints on host-switching within particular butterfly taxa tend to occur for nodes in the host and butterfly phylogenies that are deeper than those used to build these regionalizations (species; for example, subfamilies within the butterfly Pieridae feed on multiple plant orders, but host use is more conserved within subfamilies; [62,64]). Thus, while the codivergence of butterflies and their hostplants likely play a role in the correlation between their regional boundaries, shared abiotic filters such as climate and topography likely play an equivalent role in shaping how lineages in these taxa are distributed around the globe [42].

We found that the deep terrestrial boundaries between butterfly realms were consistent with those observed for terrestrial tetrapod taxa [1,12], including between the Neotropical and Holarctic realms and between the Holarctic and African and Indo-Malayan realms (figure 1), pointing to similar processes driving the divisions between realms across taxa. Rather than turnover in mean annual temperature or precipitation, butterfly realms appeared to be formed primarily by turnover in seasonality; that is, boundaries between realms occurred when regional climates shifted from relatively consistent year-round to those with more pronounced seasonal variation (figure 3). We additionally saw a strong significant effect of past climate change in driving realm boundaries. Greater historical and modern climate stability may reduce extinction rates among older lineages while simultaneously increasing speciation rates [65,66], potentially increasing the phylogenetic distinctiveness of realms and regions. On the other hand, greater seasonal variation may increase extinction rates and facilitate range expansions through adaptive migration [67,68], reducing phylogenetic beta diversity within regions. Additionally, species whose ranges abut or cross regional boundaries may have reduced adaptive capacity to overcome differences in seasonality regimes with strategies such as diapause or changes in voltinism [69–71].

Here we have presented one of the first known global biogeographic regionalization schemes for a terrestrial invertebrate taxon, including the phylogenetic and external abiotic drivers behind regional boundaries. Our results expand our understanding of how historical and contemporary factors drive the distribution of organismal lineages on the planet and provide the groundwork for future efforts to model historical dispersal, diversification and niche conservatism across taxa worldwide. Additionally, recent studies have revealed significant declines in insect abundance and diversity, but these declines are not evenly distributed across the globe or the insect tree of life [72,73]. Our results can contribute to butterfly conservation and biodiversity assessment in three key ways. First, highlighting particular global phyloregions as areas of high endemism, and of phylogenetic distinctiveness (e.g. Madagascan, the Caribbean and the Nusantaran regions) can help prioritize regions for conservation efforts [8,74]. Second, identifying ‘indicator clades’ can be useful to focus conservation efforts that preserve distinctions between regions. Finally, identifying the species and environmental factors that drive regional and realm-level divisions can provide a framework for understanding shifts in biotic regionalization and biodiversity homogenization in a changing world [9,75]. Given their extremely high diversity and wide array of complicated relationships with other organisms, studies of global insect biogeography and regionalization are much needed. We hope that studying the regionalization of a charismatic group like butterflies is an important step towards understanding how future climate and land use change will affect the processes driving global insect biogeography.

## Data Availability

We used a new set of native range polygons for butterflies created by Daru [26]. Data are available at Dryad [76]. Supplementary material is available online [77].
